# Moving Away from Ritonavir, Abacavir, Tenofovir, and Efavirenz (RATE) - Agents That Concern Prescribers and Patients: A Feasibility Study and Call for a Trial

**DOI:** 10.1371/journal.pone.0099530

**Published:** 2014-06-26

**Authors:** Amit C. Achhra, Mark A. Boyd, Matthew G. Law, Gail V. Matthews, Anthony D. Kelleher, David A. Cooper

**Affiliations:** The Kirby Institute, UNSW Australia, Sydney, New South Wales, Australia; University of Ottawa, Canada

## Abstract

**Objectives:**

Regimens sparing RATE (ritonavir, abacavir, tenofovir, efavirienz) agents might have better long-term safety. We conducted a feasibility exercise to assess the potential for a randomised trial evaluating RATE-sparing regimens.

**Design:**

Observational.

**Methods:**

We first calculated RATE-sparing options available to an average patient receiving RATE agents. We reviewed treatment history and all resistance assays from patients attending the St. Vincent’s Hospital (Sydney) clinic and receiving ≥2 RATE agents (n = 120). A viable RATE-sparing regimen with 2 or 3 fully-active agents was constructed from the following six ‘safer’ agents: rilpivirine or etravirine; atazanavir; raltegravir; maraviroc; and lamivudine. Activity for each drug was predicted as 1 (full-activity), 0.5 or 0 (no activity) using the Stanford mutation database. The utility of maraviroc was calculated assuming both maraviroc activity and inactivity where unknown. The analysis was restricted to regimens for which supporting evidence was identified in the literature or conference proceedings. Finally, we calculated the proportion of patients in the nationally representative Australian HIV Observational Database (AHOD) cohort receiving ≥2 RATE agents (n = 1473) to measure the potential population-level uptake of RATE-sparing agents.

**Results:**

Assuming full maraviroc activity, 117(97.5%) and 107(89.2%) individuals had at least one option with 2 or 3 active RATE-sparing agents, respectively. Assuming no maraviroc activity this decreased to 113(94.2%) and 104(86.7%), respectively. In AHOD, 837(56.8%) patients were receiving ≥2 RATE agents.

**Conclusion:**

Feasible treatment switch options sparing RATE agents exist for the majority of patients. Understanding the pros and cons of switching stable patients onto new RATE-sparing regimens requires evidence derived from randomised controlled trials.

## Introduction

While the development of antiretroviral therapy (ART) has seen tremendous success, there are ongoing concerns regarding the short- and long-term safety and tolerability of some of the common contemporary ART drugs. These agents and their associated concerns include: (i) Ritonavir (booster dose): long-term inhibition of CYP450, tolerance (diarrhoea) and dyslipidemia [1]; (ii) Abacavir: hypersensitivity [1], association with cardiovascular events,[1]–[3] and potency [4]; (iii) Tenofovir: long-term concern for bone and renal disease [5] and possibly heart failure [2]; and (iv) Efavirenz: neuropsychiatric and cognitive effects [6] and dyslipidemia [7], (collectively termed RATE agents in this paper). These agents are often used in combination, thereby compounding their toxicity profile.

Newer, apparently safer, more tolerable drugs continue to be approved, but the data derived from their clinical development is generally limited to their use in first-line or late salvage ART. There is a need for evidence on how agents can be innovatively combined and sequenced in order to optimally replace RATE agents and in turn achieve better long-term outcomes. A few recent small studies of novel, unconventional regimens have been reported and attest to the growing interest in more fully exploiting the opportunities offered by the growing HIV armamentarium. [8] Also, the SECOND-LINE [9], EARNEST [10] and the GARDEL [11] trials confirmed that 2 fully active agents are sufficient to achieve virological success, particularly in populations naïve to protease inhibitors (PIs).

Before planning a full scale trial evaluating RATE-sparing agents, we conducted a preliminary feasibility study to identify key metrics needed for planning such a trial. We had two key objectives: (i) in an average patient receiving ≥2 RATE agents, how many feasible RATE-sparing regimen options containing 2 or 3 fully-active agents are available if they were to switch?; and (ii) what proportion of HIV+ individuals at the population level receiving successful ART have ≥2 RATE agents as components of their regimen and might be expected to benefit from RATE-sparing options?

**Table 1:** Patient characteristics in the Hospital Database (n = 120).

Characteristics	N(%)
**Gender- Male**	113(94.2)
**Age as at June 2013, mean(SD) years**	43(9.4)
**Race- Caucasian**	114(95)
**HCV positive**	13(10.8)
**Year of HIV diagnosis, Median (IQR)**	2001(1991–2006)
**Transmission mode**	
MSM	83(69.2)
Other	13(10.8)
Unknown	24(20)
**Cumulative duration of ART in years, median (IQR)**	8.2(4.4–12.9)
**History of mono/dual therapy exposure**	19(15.9)
**Class exposure ever**	
PI	71 (59.2)
NNRTI	92 (98.9)
Integrase inhibitor	21(17.5)
CCR5 or other	6(5)
**Class resistance ever** *	
PI	20 (16.6)
NRTI	28 (23.3)
NNRTI	18(15)
All 3 classes	13(10.8)
**Treatment experience** **	
First line without substitutions	42(35.3)
First line with substitutions	43(36.1)
Second line or beyond	34(28.3)
**Maraviroc activity based on tropism test**	
Active	19(15.9)
Inactive	27(22.5)
Test unsuccessful***	9(7.5)
Unknown	65(54.2)
**Five most common regimens**	
TDF+FTC+EFV	44 (36.7)
TDF+FTC+ATV/r	22(18.3)
TDF+FTC+DRV/r	10(8.3)
ABC+3TC+EFV	9(7.5)
TDF+FTC+DRV+RTV+RAL	8(6.7)
**Sub-optimal activity score to agents of interest** ***	
**Rilpivirine**	
*-Score = 0.5*	7(5.8)
*-Score = 0*	6(5)
**Etravirine**	
*-Score = 0.5*	11(9.1)
*-Score = 0*	2(1.7)
**Atazanavir**	
*-Score = 0.5*	1(1.8)
*-Score = 0*	20 (16.7)
**3TC/FTC**	
*-Score = 0.5*	4(3.3)
*-Score = 0*	19(15.9)

*defined as high-level resistance to at least one agent from the class.

**defined as follows: First-line: no known history of resistance to any agent, and maximum change of 1 class of drugs. Second-line: history of resistance to any agent ever or major substitutions of >1 class.

***Test unsuccessful counted as unknown in the analyses.

****intergrase gene mutations had not been tested, therefore raltegravir assumed to be 1 in all patients. See text for maraviroc activity assumptions.

NOTE: ABC  =  abacavir, TDF  =  Tenofovir, 3TC  =  lamivudine, FTC  =  emtricitabine, ATV/r  =  ritonavir boosted atazanavir, DRV/r  =  ritonavir boosted darunanavir, RAL  =  raltegravir.

## Methods

### Study setting, population and analyses

#### Analysis of the St. Vincent’s Hospital’s database to calculate RATE-sparing options

For objective(i), we conducted a detailed review of the individual records of the patients attending the St. Vincents Hospital’s ambulatory care clinic (the Hospital database). This database was chosen as it provided access to detailed treatment history, tropism test, all archived resistance mutations and clinical history available in each patient. The retrospective study on the St. Vincent’s Hospital’s database was approved by the St. Vincent’s Hospital Human Research Ethics Committee, Sydney, Australia. Informed consent from participants was not obtained for this study. Requirement for informed consent for this study was waived by the above mentioned Ethics Committee. All of the data generated was made available in de-identified format to the study team.

**Table 2:** Number of treatment options under various scenarios in the Hospital database.

	Options with score of 2			Options with score of 3		
Assumption forMaraviroc use	Mediannumberof options(IQR)	Most common numberof options (%)	Number ofindividualswith zerooptions (%)	Mediannumber ofoptions(IQR)	Mostcommonnumberof options (%)	Number ofindividualswith zerooptions (%)
If R5 tropism assumed inthose unavailable	12(7–12)	12(62.5)	3(2.5)	10(4–10)	10(62.5)	13(10.8)
If R5 tropism not assumed inthose unavailable	7(7–7)	7(64.2)	7(5.8)	4(4–4)	4(64.2)	16(13.3)
**Number of treatment options for which there was some support in the literature under various scenarios in the Hospital database**						
If R5 tropism assumed inthose unavailable	5(3–5)	5(61.7)	3(2.5)	7(3–7)	7(62.5)	13(10.8)
If R5 tropismnot assumed inthose unavailable	3(3–3)	3(65.0)	7(5.8)	3(3–3)	3(64.2)	16(13.3)

We analysed the data from all patients enrolled in clinics under the following investigators: MAB, ADK, GM and DAC. Patients had to be receiving ≥2 RATE agents, in active follow-up (last visit within one year of March 2013), virologically suppressed (<200 copies/mL measured on at least 2 occasions more than 7 days apart) and not known to be hepatitis-B infected. We reviewed patient demographics, treatment history and resistance and tropism assay reports. Given that the resistance assay has been standard of care in Australia for some years, most patients were expected to have had one performed at the time of virological failure.

We defined a new regimen option as a RATE-sparing regimen constructed from a pool of the following six agents approved for use in Australia: rilpivirine or etravirine (cannot be used together); atazanavir (cannot be used with rilpivirine or etravirine because of possible unfavourable drug-drug interactions); raltegravir; maraviroc; and lamivudine. These agents were chosen for their proven efficacy and good safety profile. Also, from the PI class, only atazanavir is approved for use without a ritonavir booster. We limited consideration to raltegravir as the only integrase inhibitor available in Australia in the period we performed this analysis, but it could can be potentially be replaced by other integrase-inhibitors entering the market which will only increase the number of possible RATE sparing combinations available.

To predict drug activity we entered all recorded patient mutations (including archived results) in the Stanford mutation database version-6.3.0 (June 2013) [12]. The predicted activity was scored as 1 (susceptible or potential low-level resistance), 0.5 (low-level/intermediate resistance) and 0 (high-level resistance). Integrase inhibitor mutations were not available, but given that most patients had only been exposed to raltegravir in their most recent regimen (with complete virological suppression), full activity was assumed. The HIV tropism test for predicting maraviroc activity had been performed at the discretion of the attending physician as the part of routine care using V3 Loop DNA assay. [13] A feasible regimen ‘option’ could either have a total score of 2 or 3 (i.e. containing 2 or 3 fully active agents respectively). Since the HIV tropism test was unavailable for the majority, options were calculated assuming both maraviroc activity = of 1(full activity) or 0 (where activity unknown).

**Table 3:** Characteristics of those receiving ≥2 RATE agents and <2 RATE agents in AHOD.

Characteristics	Receiving ≥2 RATEagents (n = 837)	Receiving <2 RATEagents (including no RATE agents)(n = 636)
	N(%)	N(%)
**Gender- Male**	790(94.4)	598(94)
**Age (mean± SD) years**	50.1(±10.4)	52.3(±11.3)
**HCV positive**	87(11.5)	53(9.2)
**Cumulative duration of cART** **in years (median IQR)**	10.7(4.1–15)	13.1(5.4–15.1)
**History of mono/dual therapy** **exposure**	399(47.7)	346(54.4)
**History of mono/dual therapy** **exposure for at least 30 days**	318 (38.0)	236(37.1)
**Class exposure ever**		
PI	570(68.1)	374(58.8)
NNRTI	722(86.3)	546(85.9)
Integrase inhibitor	116(13.7)	201(31.6)
CCR5	10(1.2)	12(1.9)
**Total number of classes** **exposed to**		
Median	3	3
Mean	2.7	2.8
**Exposure to >2** **classes**	466 (55.7)	371(58.3)
**RATE agents**		
Ritonavir	466(55.7)	102(16.0)
Abacavir	246(29.4)	203(31.2)
Tenofovir	644(76.9)	265(41.7)
Efavirenz	386(46.1)	10(1.6)
None	0(0)	56(8.8)

**Note:** RATE =  ritonavir, abacavir, tenofovir and efavirenz. cART =  combination antiretroviral therapy with at least 3 drugs.

Finally, since most of the RATE-sparing regimens were unconventional, we conducted a review of the peer-reviewed literature (using MEDLINE database) and major conference presentations to identify regimens for which there was at least some empirical support (defined as at least one study showing at least 24 or 48 week virological efficacy >80%). The search was conducted using key words for each of the agents in consideration (or their respective ART classes) using Boolean operators. We restricted the search to articles in English language published in the year 2006 or later. We included studies on regimens of interest regardless of study phase, number of patients or availability of a comparator arm.

#### Analysis of the AHOD cohort to quantify use of RATE agents in the population

For objective(ii), we analysed the data from the Australian HIV Observational Database (AHOD) cohort. The AHOD is an observational cohort study of HIV-positive individuals attending specialised general practitioner sites, sexual health clinics and tertiary referral centres throughout Australia. This study has been ongoing since 1999, and currently has 27 sites throughout Australia (including St. Vincent’s Hospital Sydney). The AHOD study has been approved by the Human Research Ethics Committee of the University of New South Wales, Sydney, Australia, and all other relevant institutional review boards. Written informed consent was obtained from participating individuals in the AHOD study. The details of the AHOD study design have been published elsewhere [14].

We identified patients in the AHOD who were in active follow-up (documented visit within one year of March 2013 data transfer), receiving ART, virologically suppressed (HIV RNA <200 copies/mL) at their last visit, and not known to be co-infected with hepatitis B (HBsAg negative). Our aim was to identify the proportion of such patients receiving ≥2 of RATE agents at their last follow-up visit and describe their demographic and treatment-history characteristics.

All analyses were performed using Ms Excel (Microsoft) and STATA ver. 12 (STATA Corp, Texas, USA).

## Results

### The St. Vincent’s Hospital’s database

A total of 120 patients from the Hospital database matched the selection criteria and were included in analyses. Table-1 describes their characteristics. They had been receiving ART for a median of 8 years (IQR: 4.4–12.9) and about 17% had exposure to integrase inhibitors. An HIV tropism test for predicting maraviroc activity was unavailable or unsuccessful in 54.2% and 7.5% of patients, respectively and 19 (15.9%) patients had full expected activity of the maraviroc. All HIV tropism tests were available within the last year (earliest one was in March-2012). About 16% of patients had no expected activity of either unboosted atazanavir or lamivudine.

Table-2 describes the number of available RATE-sparing regimen options under various scenarios. Assuming maraviroc activity = 1 where unknown, 117 (97.5%) and 107 (89.2%) individuals had at least one option with a score of 2 or 3, respectively. This decreased to 113 (94.2%) and 104 (86.7%), respectively, on assuming maraviroc inactivity where unknown.

The literature review indicated that direct or indirect support was available for many novel regimens, mainly in the form of small pilot studies [8] (key studies summarised in Table-S1 [15]–[30]. These included studies of the following two-drug regimens: raltegravir+maraviroc [16]; raltegravir+atazanavir [17]–[22] as well as with other protease inhibitors [31]–[33]; maraviroc+atazanavir [23], [24], lamivudine +atazanavir (evidence available for only boosted atazanavir) [25], [34], and raltegravir+etravirine [26], [28], [29]. It can be reasonably assumed that adding a third compatible agent to the above two-drug regimens would offer at least equal efficacy (e.g. adding maraviroc to raltegravir+etravirine [28]–[30]). The only exception might be in the case of raltegravir combined with other agents with a relatively low genetic barrier to resistance (e.g. raltegravir with rilpivirine and lamivudine; or raltegravir with rilpivirine and maraviroc) which were not included in the analyses.

Table-2 also describes a number of available RATE-sparing regimen options for which we could identify supporting evidence. Although the median number of available regimen options decreased in all of the scenarios, there was no appreciable change in the proportion of patients without any option remaining.

### The AHOD cohort

Of the 1473 eligible patients in AHOD, 837 (56.8%, 95% confidence interval: 54.2%–59.4%) were receiving ≥2 RATE agents. Table-3 provides the characteristics of patients in AHOD who were receiving ≥2 RATE agents. Patients with ≥2 RATE agents had been receiving ART for a median of 10.7 (interquartile range (IQR): 4.1–15) years, 38% with a history of mono/dual therapy exposure in the past; they had been exposed to a median of 3 classes of drugs; 13.7% had been exposed to integrase inhibitors. The most common RATE agent used was tenofovir (76.9%) followed by ritonavir (in boosting dose) (55.7%).

## Discussion

Our analysis of patients in one hospital-based clinic suggested that most patients receiving ≥2 RATE agents would be expected to have at least one viable RATE-sparing regimen switch option containing 2 or 3 fully-active agents This remained the case when we restricted our assessment to only those regimen options which have shown promise in publicly presented studies. In our analysis of the AHOD we found that up to 57% of HIV-positive individuals under treatment in Australia currently have ≥2 RATE agents in their regimen and might benefit from a RATE-sparing option. These findings suggest that there exits the potential to perform randomised trials to evaluate RATE-sparing regimens. Such trials would provide valuable guidance on how best to combine and sequence ART agents to maximise patient safety and preserve future treatment options.

Most of the regimens proposed and considered as ‘viable’ in this study have not been rigorously tested in clinical trials and might be regarded as unconventional, and perhaps as possessing a lower genetic barrier to the selection of resistance. However, there has been growing interest in testing novel combinations of ART agents, which exclude nucleoside(tide) and older non-nucleoside reverse transcriptase inhibitors (N(t)RTIs and NNRTIs, respectively) as well as ritonavir (booster dose) [35]. The SECONDLINE [9] and EARNEST [10] trials suggest that a strategy of carefully selecting 2 fully-active agents is likely to be successful. Other studies suggest that raltegravir combined with one or two other active agents (e.g. a protease inhibitor or maraviroc or a second generation NNRTI) is a viable option in patients, even those with extensive treatment experience. [28], [30], [36] In the NEAT001 trial in ART-naïve individuals, the combination of raltegravir+ boosted-darunavir performed well although less well in those with baseline VL>100,000 copies/mL. [37] In the 48-week LATTE phase-2b trial, the combination of a doultegravir-analogue with rilpivirine performed equally well in comparison to conventional EFV-containing triple therapy in maintaining virological suppression after induction with conventional NtRTI-containing triple therapy. [15] One small study (Roc N Ral) study [38], the virological failure rate in the raltegravir+maraviroc arm was high (21%). Of note, the tropism was analysed using Geno2Pheno (rather than phenotypic assay) and failure was linked to adherence <80%.

A few limited studies on unboosted atazanavir and raltegravir show encouraging results in terms of virological success and safety profile, although hyperbilirubinemia might be an issue with unboosted atazanavir containing regimens, especially if atazanavir is used at a dose of 300 mg twice-daily. [18] However, after>10 years in clinical use atazanavir is not known to be associated with adverse long-term outcomes, atazanavir at 200 mg twice daily provides adequate pharmacokinetic coverage and discontinuation for hyperbilirubinaemia is relatively uncommon. [39] Similarly, combining boosted-atazanavir with maraviroc has shown promising results (comparable virological response at 48 weeks to tenofovir + emtricitabine + boosted-atazanavir regimen) [24]. Further, replacing boosted atazanavir with un-boosted atazanavir (as considered in this study) in virologically suppressed individuals appears to be safe and effective. [40].

In one small single-arm trial, ART-naïve patients achieving virological suppression with a four drug combination of tenofovir, emtricitabine, maraviroc and raltegravir, safely stopped the N(t)RTI component after 24 weeks without any failure 24 weeks after stopping the N(t)RTIs. [16] In another study of patients who had experienced triple-class failure, a regimen of 3 fully-active agents (raltegravir, maraviroc and etravirine) demonstrated 96% virological efficacy and improvement in lipid profiles at 96 weeks of follow-up [29]. Finally, a dual drug combination of raltegravir and etravirine in treatment experienced patients without past NNRTI failure has shown promising results. [26] Of note, emergence of integrase mutations in dual therapy regimens containing raltegravir tend to occur in those with HIV RNA>100,000 copies/mL at the time of switch. [28], [41] Overall, these studies support the notion that 2 or 3 fully-active pharmacologically compatible agents result in robust virological response in carefully selected patients.

**Figure 1:**
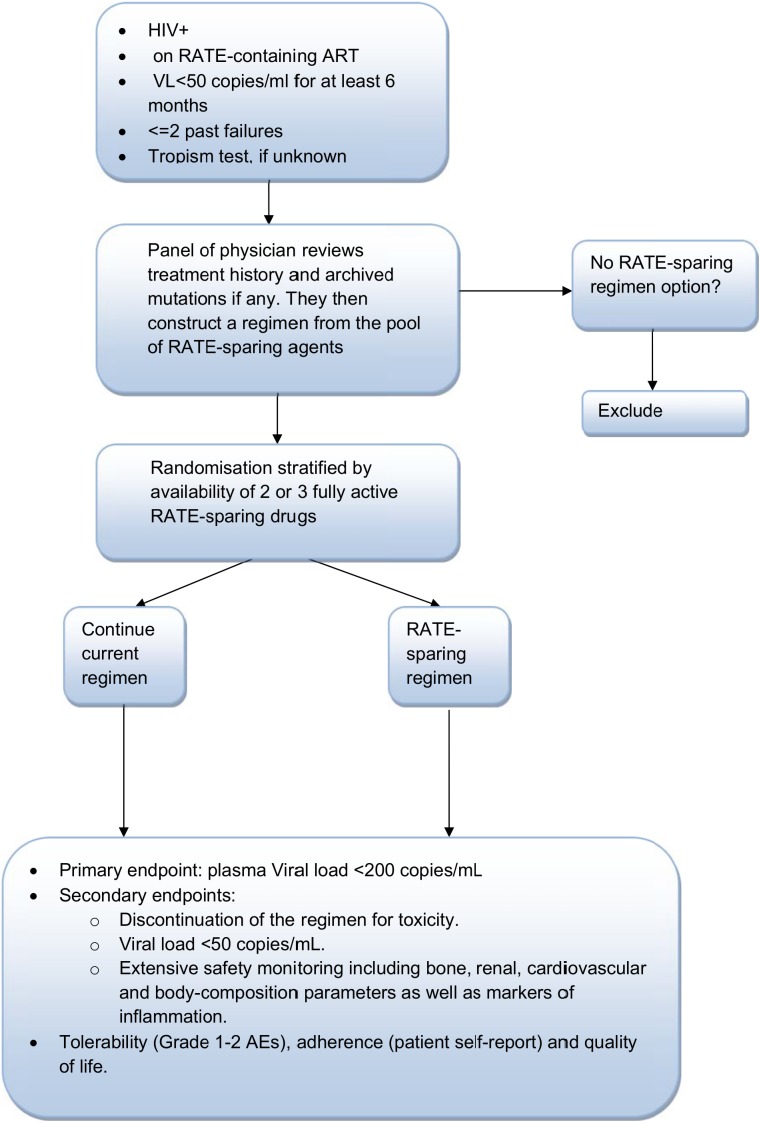
Proposed trial for evaluating RATE-sparing options.

Despite these observations there is a clear need for trials to rigorously evaluate RATE-sparing regimens, not only to demonstrate their virological success but also their expected greater tolerability and safety. Given the concern for possible resistance emergence in those with high viral loads, trials could initially focus on virologically suppressed patients with no or limited history of treatment failure and high expected adherence. The scheme of a proposed trial design is represented in Figure 1. The end-points include virological suppression as well as extensive safety monitoring. Ideally such a trial should have hard clinical end-points to demonstrate safety. At the very least it would need to be a 96 week study monitoring metabolic, renal, bone and body composition parameters. Drop-out or premature switching in the control arm would be handled by intention-to-treat follow-up and analysis to minimise bias.

The following additional/alternative trial strategies would be worth considering: (i) enrolling patients thought to be at a high risk of a chronic disease such as cardiovascular disease and therefore most likely to benefit from the trial. [42] This may be relevant for some drugs, for example abacavir. However, drugs such as ritonavir are undesirable even to low-risk patients; (ii) providing physicians with a menu of 3–4 reasonable RATE-sparing regimens so that the trial has enough power to make some conclusions about individual regimens as well as about the overall strategy of switching. Given that a vast majority of our patients had >0 activity for most of the RATE-sparing agents (except about 16% fully resistant to atazanavir and lamivudine eachTable-1), it is clear that a list of 3–4 RATE-sparing regimen options would be available to most patients.

Our study has limitations. Firstly, it was a retrospective survey of patient data. It analysed data from patients attending a single large tertiary hospital centre in Sydney (for objective i). It is possible that more complicated or difficult to manage patients may have been selectively referred to this centre, resulting in lesser generalizability of our findings. However, this may have only resulted in underestimation of the actual number of options available to patients in general. Also, patient characteristics in the Hospital database were broadly similar to those in AHOD, suggesting minimal selection bias. Second, the Hospital database may not have accurately captured comorbidities and concomitant medication data which may impact the number of suitable options available to a patient. However, the agents considered for treatment options are known to have only few serious drug-drug interactions or contraindications. Our assessment of the recorded data on comorbidities and concomitant medications did not affect our conclusions. We therefore do not believe this limitation would seriously undermine the number of options available. We did not consider use of atazanavir and etravirine together as an option (due to drug-drug interactions), though a recent study suggests that the use of a higher dose of atazanavir in such a combination may not be necessary. [43] Allowing these two agents in combination would increase the number of options available. Finally, many NRTI-sparing regimens may require twice daily dosing frequency and ≥1–2 pills/day. This may impact the adherence. However, both maraviroc (with a boosted atazanavir) and etravirine have pharmacokinetic and clinical data supporting once-daily dosing [24], [44], and raltegravir is being studied in a 1200-mg once-daily formulation. Some RATE-sparing regimens might well become available with fewer pill burden/infrequent dosing.

In summary, our study suggests that most patients using RATE agents have viable RATE-sparing switch options which include 2 or 3 fully active agents in the switch regimen. The use of RATE drugs is common and a significant proportion of HIV-positive individuals might be expected to benefit from such options. There is a need for fully powered randomised trials to rigorously evaluate this strategy in order to optimise long-term patient outcomes.

## Supporting Information

Table S1 **Summary of regimens supported by the literature.**
(DOCX)
