# Supplementary material for: Moving Away from Ritonavir, Abacavir, Tenofovir, and Efavirenz (RATE) - Agents That Concern Prescribers and Patients: A Feasibility Study and Call for a Trial
Source: PLoS One. 2014 Jun 26;9(6):e99530. doi: 10.1371/journal.pone.0099530 (PMC4072535; doi:10.1371/journal.pone.0099530)
Supplement: Table S1 — Summary of regimens supported by the literature. (DOCX) [file pone.0099530.s001.docx]

**Table-S1: Summary of regimens supported by the literature.**

| **Regimen** | | **Author, Trial Name/Year/Published?** | | | **Design and comparison** | | **N** | **Follow-up (weeks)** | | **Results** | |
| --- | --- | --- | --- | --- | --- | --- | --- | --- | --- | --- | --- |
| **Two drug regimens** | | | | | | | | | | | |
| **RAL + Integrase inhibitor (doultegravir analogue)** | | | Margolis et al/ LATTE[15]/2014/No | Phase 2b randomised study after induction therapy, comparing (i) GSK744+ RPV (ii) conventional EFV+ NRTI based regimen | | 60 in each arm | | 48 | | 96% and 94% efficacy in arms (i) and (ii) respectively. Higher rate of mutations in arm (ii). More neuro-psychiatric events in arm (ii) | |
| **RAL+MVC** | | | Cotte et al, No Nuc No Boost[16] / 2013/ No | Single-arm exploratory trial of patients receiving RAL+MVC | | 10 | | 48 | | No virological failures (>50 copies/mL). No serious adverse event | |
| **RAL+RPV** | | | **-** |  | |  | |  | |  | |
| **RAL+ATV** | | | Ward et al[17]/2013/No | Observational. Switching for toxicity concerns to a RAL+ 1 or 2 agents, most commonly on RAL+ATV/r with or without ETV or MVC | | 62 | | 168 | | 92% virologically suppressed. 3 of 15 on dual therapy had to add third agent for low-level viremia | |
|  |  |  | Kozal et al, SPARTAN [18]/2012/Yes | RCT pilot study of (i) ATV+RAL; (ii) ATV/r+ TDF+FTC | | (i) 63; (ii) 31 | | 24 | | 74.6% in arm(i) and 63.3% in arm(ii) had viral load <50 copies/mL. 4/6 failures in arm(i) had RAL mutations. 20% incidence of grade-4 hyperbilirubenimia in arm(i). | |
|  |  |  | Carey et al SPARTA[19]/ 2012/ Yes | Patients receiving ATV/r randomized to: (i) ATV/r (300/100 mg respectively once daily) +RAL (800mg once daily); (ii) ATV (300mg twice daily)+ RAL (400mg twice daily) | | 25 | | 76% in follow-up for 48 weeks | | Both agents pharmacologically compatible. All patients remained virologically suppressed | |
|  |  |  | Cordery et al[20]/ 2010/ Yes | Observational study of patients receiving RAL+ATV (unboosted) | | 20 | | 72 | | Only 1 (5%) failure | |
|  |  |  | Allavena et al[21]/2009/Yes | Observational. Patients switching for toxicity concerns to a PI/r+ RAL. | | 29 | | 48 | | 100% virologically suppressed | |
|  |  |  | Gupta et al[22]/2009/Yes | Case series of patients receiving RAL with unboosted ATV | | 6 | | 25-82 weeks | | No virological failures (>50 copies/mL). No serious adverse event | |
|  | | | Wilkin et al[23] 2009/Yes | Virologically suppressed patients switched to ATV/r alone | | 34 | | 48 | | 30 (88%) virologically suppressed (<200 copies/mL). No major PI mutations identified at failure. | |
| **RAL+3TC** | | | - |  | |  | |  | |  | |
| **MVC+ETV** | | | - |  | |  | |  | |  | |
| **MVC+ATV** | | | Mills et al[24], A4001078/ 2013/Yes. Also see Wilkin et al[23] (above) | RCT Phase 2b pilot, comparing (i) MVC +ATV/r; (ii) TDF+FTC+ATV/r | | 121 | | 48 | | 75% in arm (i) and 84% in arm(ii) had viral load <50 copies/mL. More hyperbilirubenmia in arm(i). Nine in arm(i) and 3 in arm(ii) had low-level viremia after virological suppression. | |
| **ETV+3TC** | | | - |  | |  | |  | |  | |
| **RPV+3TC** | | |  |  | |  | |  | |  | |
| **ATV+3TC** | | | Di Giambenedetto et al[25], AtLas/2013/Yes | Single arm pilot study. Virologically suppressed patients receiving ATV/r+N(t)RTIs switched to ATV/r +3TC | | 40 | | 48 | | Only 1 virological failure. Total 4 (10%) discontinued the regimen | |
|  | | | Also see Wilkin et al[23] (above) |  | |  | |  | |  | |
|  | | | Also, Cahn P et al GARDEL study team[11] | Phase-III, randomised, controlled trial comparing (i) LPV/r + 3TC and (ii) LPV/r + 2 NRTIs in ART-naive patients | | (i) 214, (ii) 202 | | 48 | | Arm (i) non-inferior to (ii) in virological efficacy (<50 copies/mL). Fewer discontinuations in arm (i), though most patients in arm(ii) received thymidine analogues. | |
| **RAL+ETV** | | | Calin et al[26]/2013/No | Observational: Virologically suppressed patients switching to RAL+ETV regimen | | 91 | | 48 | | 93% had viral load <50 copies/mL. 4/5 with virological failures had past NNRTI mutations. 3 patients had RAL mutations | |
|  | | | Monteiro et al[27] | Observational: Virologically suppressed patients switching to RAL+ETV regimen | | 25 | | 48 | | 84% by intention-to-treat and 91% by per-protocol analysis virologically suppressed (<50 copies/mL) | |
| **MVC+3TC** | | | - |  | |  | |  | |  | |
| **MVC+RPV** | | | - |  | |  | |  | |  | |
| **Three drug regimens** | | | | | | | | | | | |
| **RAL+MVC+ETV** | Ward et al[17](see above) | | |  | |  | | |  | |  |
|  | Imaz et al[28]/2011/ Yes | | | Observational: Salvage regimen of at least three active agents from DRV, ETV, RAL and MVC, with or without N(t)RTIs. | | 122 | | | 48 | | 78% virologically suppressed (equal in both arms). Higher baseline viral load associated with worse outcomes. |
|  | Nozza et al[29]/ 2011/ Yes | | | Observational: Salvage regimen of RAL+MVC+ETV | | 28 | | | 96 | | 96% virologically suppressed (<50 copies/ml) |
|  | Tashima et al[30], OPTIONS/ 2013/ No | | | Phase-3/4 RCT, non-inferiority study: (i) NRTI-omitting optimised regimen vs. (ii) NRTI-including optimised regimen in triple-class experienced failing patients | | (i)179; (ii) 181 | | | 48 | | Similar virlogical outcomes in both arms. No differences in grade 3/4 events. Higher mortality in arm(ii). |
| **RAL+ATV+3TC** | No direct evidence, but evidence on use of RAL+ATV dual therapy (see above) | | |  | |  | | |  | |  |
| **MVC+ATV+3TC** | No direct evidence, but evidence on ATV/r as mono-therapy as well as on MVC+ATV (see above) | | |  | |  | | |  | |  |
| **ETV+3TC+RAL** | No direct evidence, but evidence on RAL+ETV dual therapy | | |  | |  | | |  | |  |
| **RPV+3TC+RAL** | - | | |  | |  | | |  | |  |
| **3TC+RAL+MVC** | No direct evidence, but evidence on RAL and MVC dual therapy (see above) | | |  | |  | | |  | |  |
| **3TC+MVC+ETV** | - | | |  | |  | | |  | |  |
| **RPV+RAL+MVC** | - | | |  | |  | | |  | |  |
| **RAL+ATV+MVC** | Please see above: Ward et al[17]; Imaz et al[28] and Tashima et al[30] | | |  | |  | | |  | |  |

**NOTE:** 3TC= lamivudine; ATV= atazanavir; ETV= etravirine; LPV/r= boosted lopinavir; MVC= maraviroc; RAL=raltegravir; /r= ritonavir in booster dose; RPV=rilpivirine. Regimens for which no evidence could be found were not included in the analyses focusing on regimens supported by the literature.
